# Expression profile analysis of 5-day-old neonatal piglets infected with porcine Deltacoronavirus

**DOI:** 10.1186/s12917-019-1848-2

**Published:** 2019-04-16

**Authors:** Jiao L. Wu, Kai J. Mai, Di Li, Rui T. Wu, Zi X. Wu, Xiao Y. Tang, Qian N. Li, Yuan Sun, Tian Lan, Xiang B. Zhang, Jing Y. Ma

**Affiliations:** 1 College of Animal Science, South China Agricultural University, Tianhe District, Wushan Road 483, Guangzhou, 510642 China; 2 Key Laboratory of Animal Health Aquaculture and Environmental Control, Guangzhou, Guangdong China; 3 Guangdong Wen’s Foodstuffs Group CO., Ltd., Guangzhou, Guangdong China

**Keywords:** Porcine *deltacoronavirus*, mRNA expression profile, Metabolism, PPAR signaling pathway

## Abstract

**Background:**

Porcine *deltacoronavirus* (PDCoV) is a novel coronavirus that can cause diarrhea in nursing piglets. This study was aimed to investigate the roles of host differentially expressed genes on metabolic pathways in PDCoV infections.

**Results:**

Twenty thousand six hundred seventy-four differentially expressed mRNAs were identified in 5-day-old piglets responded to PDCoV experimental infections. Many of these genes were correlated to the basic metabolism, such as the peroxisome proliferator-activated receptor (PPAR) signaling pathway which plays a critical role in digestion. At the same time, in the PPAR pathway genes of fatty acid-binding protein (FABP) family members were observed with remarkably differential expressions. The differential expressed genes were associated with appetite decrease and weight loss of PDCoV- affected piglets.

**Discussion:**

Fatty acid-binding protein 1 (FABP1) and fatty acid-binding protein 3 (FABP3) were found to be regulated by PDCoV. These two genes not only mediate fatty acid transportation to different cell organelles such as mitochondria, peroxisome, endoplasmic reticulum and nucleus, but also modulate fatty acid metabolism and storage as a signaling molecule outside the cell. Therefore, it can be preliminarily concluded that PPAR differential expression caused by PDCoV was mostly associated with weight loss and death from emaciation.

**Conclusions:**

The host differentially expressed genes were associated with infection response, metabolism signaling and organismal systems signaling pathways. The genes of FABP family members in the PPAR signaling pathway were the most highly altered and played important roles in metabolism. Alteration of these genes were most likely the reason of weight loss and other clinical symptoms. Our results provided new insights into the metabolic mechanisms and pathogenesis of PDCoV infection.

**Methods:**

Animal experiment, Determination of viral growth by real-time RT-PCR, Histopathology, Immunohistochemical staining, Microarray analysis.

## Introduction

*Coronaviruses* (CoVs) are single-stranded, positive-sense and enveloped RNA viruses. The *Coronaviridae* contains at least four major genera that includes *Alpha*, *Beta*, *Gamma* and *Delta coronavirus* [1]. In 2009, *Delta coronaviruses* was first discovered from a wide range of domestic and wild birds in Hong Kong [2]. And in 2012, Porcine *Deltacoronavirus* (PDCoV) was reported from rectal swabs from pigs in Hong Kong, represented by strains of HKU15–155 and HKU15–44 [3]. PDCoV identified from pigs can cause severe diarrhea, vomiting and dehydration in piglets, which are similar to the symptoms of *porcine epidemic diarrhea virus* (PEDV) and *transmissible gastroenteritis virus* (TGEV), except the mortality rate of PDCoV infection lower than those of PEDV or TGEV [4, 5]. So far, PDCoV has circulated in pigs in Hong Kong, North America, South Korea, Thailand and some provinces of Mainland China, which has resulted in huge economic losses for the global swine industry [6–8].

There have been numerous studies examining the pathogenesis and host response to PDCoV infections [9–11]. For example, the recent study showed that binding of PDCoV to an interspecies conserved site on aminopeptidase N (APN) may facilitate the direct transmission of PDCoV to nonreservoir species, including humans [12]. Although there is no relevant report illustrating that PDCoV has the possibility of cross-species transmission between animals and humans, it is significant to improve the awareness about biosecurity and imperative to research and analysis the pathogenesis of PDCoV. So far, there are no relevant studies about the expression profile of PDCoV, especially on animals. In this study, mRNA transcriptome of 5-day-old piglets responded to PDCoV infections was identified. A number of genes associated with the peroxisome proliferator-activated receptor (PPAR) signaling pathway were significantly differentially expressed. The results would provide new information regarding the metabolic mechanisms and pathogenesis of PDCoV infection.

## Materials and methods

### Cell culture and virus infection

The swine testicular cell (ST) (ATCC-CRL 1746) were kept in our laboratory and cultured in Dulbecco’s modified Eagle’s medium (DMEM, HyClone) in T-flasks (25 cm^2^) with 10% *v*/v fetal bovine serum (FBS, HyClone) at 37 °C within 5% CO_2_ incubator. Monolayer cells were washed three times with DMEM and then were infected with PDCoV CHN-GD16–05 (GenBank accession no.KY363868.1) strain at a MOI of 1.5 [13]. When ST cells developed > 80% cytopathic effects (CPE) at 36 h post-infection (hpi), virus harvested with a titer of 1.0 × 10^8.6 TCID_50_/mL. Finally, PDCoV was propagated in ST cells until sixth passage.

### Experimental design

A total of 35 piglets that were 5-day-old were purchased form a commercial farm. Rectal swabs from these piglets were collected to detect PDCoV, TGEV and PEDV by virus-specific PCRs, which indicating the negative results. Then 35 piglets were divided into two groups, the experimental group containing 20 piglets and the mock-control group including 15 pigs. Piglets in the experimental group were inoculated with PDCoV(CHN-GD16–05) with 5 ml at 1.0 × 10^8.6 TCID_50_/mL by oral administration, and weighted every day during the experimental period. Fecal samples were collected by cotton swabs from all piglets on days 0, 1, 2, 3, 4, 5, 6, 7, 14 and 21 days post-infection (dpi) and then were put into the EP tubes of 2 mL containing DMEM. The small intestine including duodenum, jejunum and ileum from 18 PDCoV-inoculated piglets were collected and examined for gross lesions at early (2 dpi), mid-onset (4 dpi) and late onset (11 dpi), with six piglets detected for each dpi. As the same time, the gene expression profiling was also analyzed for these 18 infected piglets and 15 piglets in the control group.

After the experiment, piglets was humanely euthanized after intravenous injection of 80 mg/kg body weight sodium pentobar-bital, and a complete necropsy was performed to collect tissues.

### Determination of viral growth by real-time RT-PCR

One-step real-time RT-PCR reactions were performed using ABI PRISM 7500 instrument and SYBR Green qPCR SuperMix (Invitrogen, Carlsbad, CA, USA). All samples were assayed in triplicate. Primers were selected and designed using Primer premers 5.0 to generate 160 bp real-time RT-PCR product (Table 1). The sensitivity of the qRT-PCR assay herein was able to detect approximately 2.0 × 10^1^ copies/μL of the PDCoV clone. The real-time RT-PCR gave positive results for the standard plasmid of PDCoV strain (2.0 × 10^9^ copies/μL) and negative results (Ct > 35) for other porcine viruses, including PEDV, TGEV, Rotavirus (RV), Senecavirus A (SVA), Classical swine fever (CSF), Porcine reproductive and respiratory syndrome virus (PRRSV), Picobirnavirus (PBV) and Porcine circovirus 3 (PCV3).

**Table 1:** Primers and probe for PDCoV detection

primers	Sequence(5′-3′)	amplicon
qPDCoV-F	TGGCTGATCCTCGCATCATGG	155 bp
qPDCoV-R	GAGCGCATCCTTAAGTCTCTC	

### RNA extraction, library construction and sequencing

Samples of the jejunum closed to the ileum were collected from 18 experimental piglets for Microarray analysis. Nucleic acid was extracted by the Trizol Kit (Promega, USA) following by the manufacturer’s instructions and then was treated with RNase-free DNase I (Takara Bio, Japan) for 30 min at 37 °C to remove residual DNA and obtain RNA. The RNA quality was verified using a 2100 Bioanalyzer (Agilent Technologies, Santa Clara, CA) and also checked by RNase free agarose gel electrophoresis. Next, Poly (A) mRNA was isolated using oligo-dT beads (Qiagen). All mRNA was broken into short fragments by adding fragmentation buffer. First-strand cDNA was generated using random hexamer-primed reverse transcription, and then primitive fragmented mRNA was digested by RNase H, followed by the synthesis of the second-strand cDNA. The second-strand cDNA was added end reparation poly (A) and ligated to sequencing adapters. The second-strand cDNA with bases “U” was digested by the enzyme UDG. Finally, the first-strand cDNA was enriched by PCR to construct the final cDNA library. The cDNA library was sequenced on the Illumina sequencing platform (IlluminaHiSeq 2000) using the paired-end technology by Personal Biotechnology Co., Ltd. (Shanghai, China). A Perl program was written to select clean reads by removing low quality sequences. These included the samples with quality scores < Q20, and reads containing adaptor sequences. The sequencing data is available from the NCBI Short Read Archive (SRA) (http:// www.ncbi.nlm.nih.gov/sra).

### Histopathology

Tissue samples of duodenum, jejunum and ileum from the experiment group and the control group in 2 dpi, 4 dpi and 11 dpi were routinely fixed in 10% formalin and processed by the following steps, which included dehydrating in graded ethanol, embedding in paraffin, sectioning to 5-μm sections, and final staining with hematoxylin and eosin (H&E). Slides were examined by conventional light microscopy.

### Immunohistochemical staining

The study of Chen et al. introduced the preparation method of monoclonal antibodies and the anti-PDCoV-N protein was used as primary antibody in this article [14]. The slides was processed according to previous studies [6, 11]. Slides were then dehydrated, cleared with xylene and sealed with cover slips. Finally, the slides were observed under an Olympus BX40 fluorescence microscopy.

### Microarray analysis

Feature Extraction software (Version10.7.1.1, Agilent) was used to analyze array images to get raw data. Genespring (Version13.1, Agilent) was employed to finish the basic raw data analysis. The raw data was normalized using the quantile algorithm. Probes possessed at least 100% of the values in any one out of all conditions were flagged as “Detected” and chosen for further data analysis. Differentially expressed genes were then identified through fold change as well as *P* values calculated using the Students t-test. The threshold set for up- and down-regulated genes was a fold change ≥2.0 and a P value≤0.05 compared with data of piglets in control group.

To confirm the microarray data, six genes were selected for the verification by real-time PCR (see Table 1 for primer sequences). One-step real-time qRT-PCR reactions were performed using One Step PrimeScript RT-PCR kit (Takara) using an ABI PRISM 7500 instrument with the following steps: reverse transcription at 42 °C for 10 min, denaturation at 95 °C for 10 min and 40 cycles at 95 °C for 15 s, 60 °C for 30 s, 72 °C for 30 s and melting curve analysis. All samples were assayed in triplicate and relative gene expression was calculated as the previously described method [15].

## Results

### Clinical assessment

**Fig. 1:**
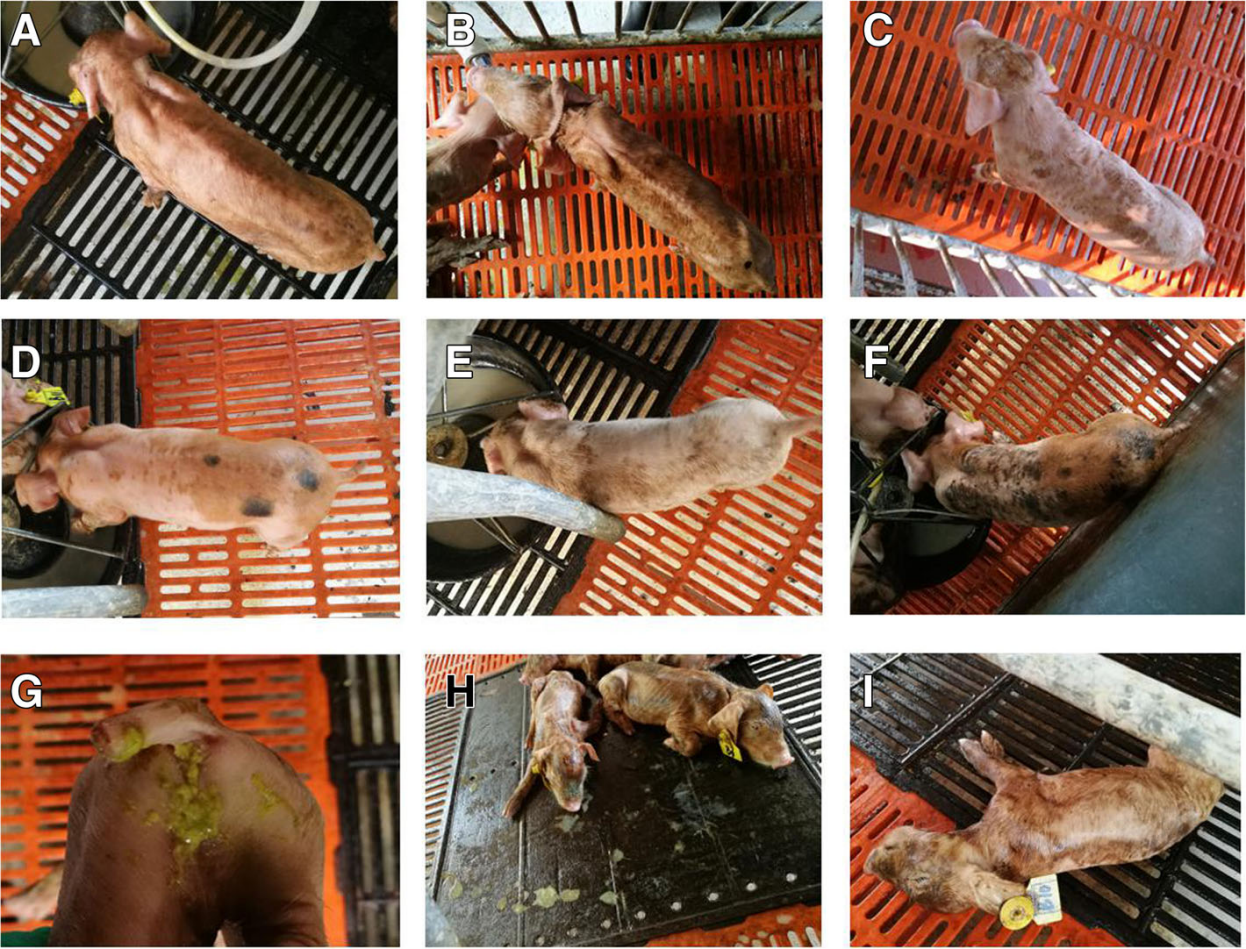
The clinical symptoms were showed in Fig. 1. **a** pig’s appearance of the experimental group in 2 dpi; (**b**) pig’s appearance of the experimental group in 4 dpi; (**c**) pig’s appearance of the experimental group in 11 dpi; (**d**) pig’s appearance of the control group in 2 dpi; (**e**) pig’s appearance of the control group in 4 dpi; (**f**) pig’s appearance of the control group in 11 dpi; (**g**) watery diarrhea in pig of the experimental group; (**h**) pig vomiting; (**i**) the pig death

**Fig. 2:**
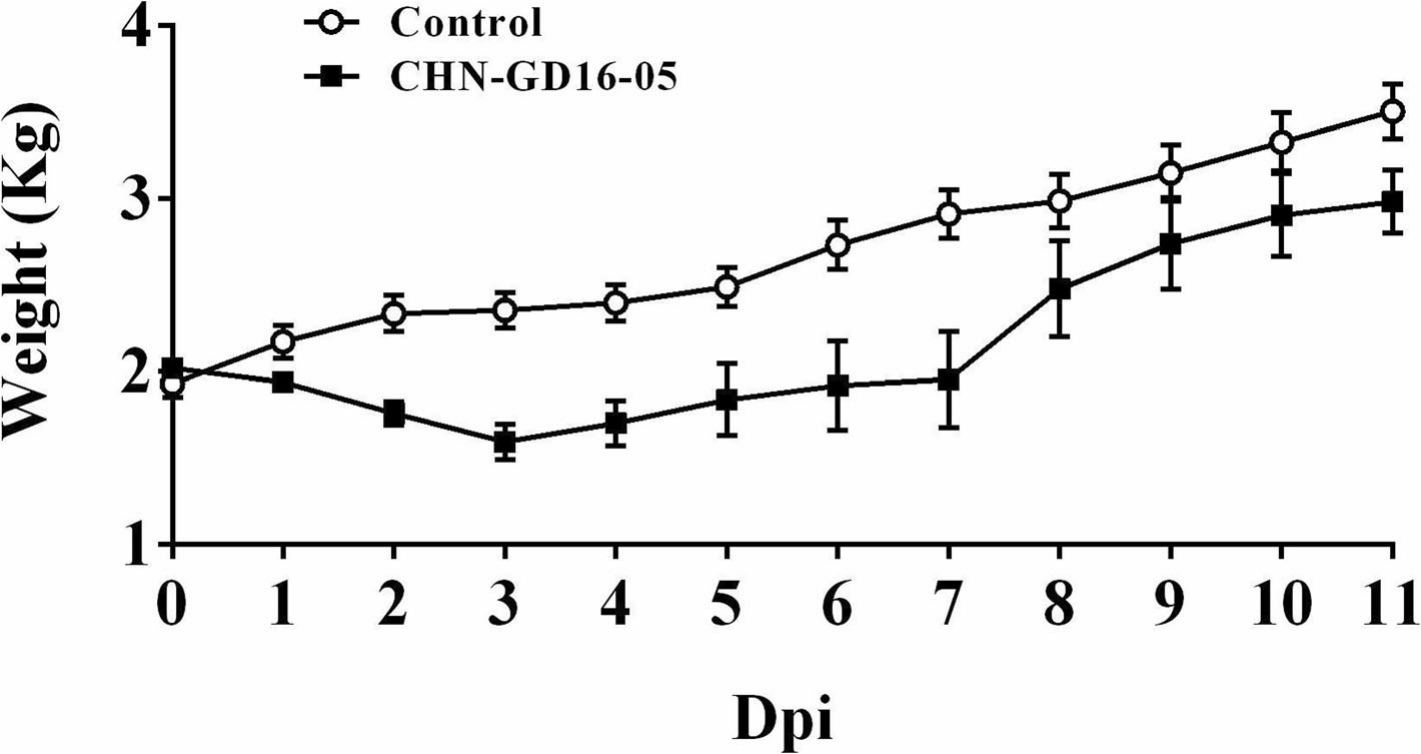
Trend of weight gain of piglets after PDCoV-inoculated. The weight gain of piglets in the experiment group showed decline at first, then slowly increased, and finally increased sharply. The period of weight loss is from 1 dpi to 3 dpi, which was from the early stage to the peak stage and finally piglets’ weight reached the lowest average of only 1.55 kg in 3 dpi

In PDCoV-inoculated pigs, 7 of 20 pigs had soft feces during 2–4 dpi, 6 of 20 pigs had watery diarrhea and 3 piglets dead during 5–7 dpi. 3 of 20 piglets recovered at 8 dpi. The clinical manifestation was most obvious at 2 dpi, including the weight loss and the appetite decrease (Fig. 1a and c). Some susceptible piglets’ anus began to become red and swollen and even excrete watery feces. The third to fourth days after PDCoV-inoculation were the peak of the clinical manifestations for all experimental piglets, which included the sudden drop in weight, emaciated body with visible ribs, anorexia, always lying down, and anus turning red, swollen and moist (Fig. 1b and g). Feces appeared yellow watery and even some piglets developed convulsions, vomiting and dead (Fig. 1g, h and i). During 8–11 dpi, the surviving animals started to recover, for example, their appetites and weight increased to the average level of the control group (Fig. 1e and f) (Fig. 2).

### The detection of virus distribution by qRT-PCR

**Fig. 3:**
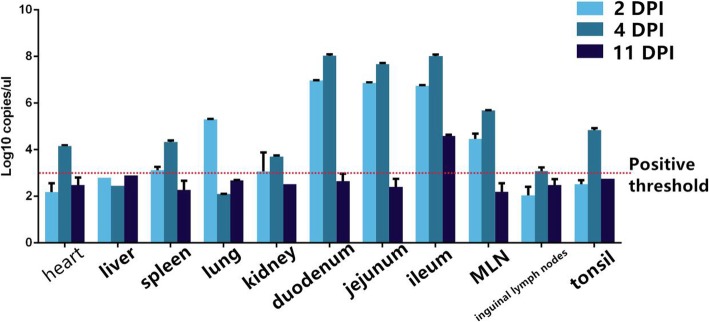
Statistical chart of qRT- PCR detection in the PDCoV-inoculated piglets. Tissue samples of inoculated piglets were collected in sterile method in 2 dpi, 4dpi and 11 dpi. Determination and statistics of virus content in various organs using established detection methods. The statistical results are shown in the figure

Virus distribution in the diseased piglets was examined at the early, middle and late stages by PDCoV-specific PCR. Virus was detected from all duodenum, jejunum, and ileum at the early stage with average 7log10 RNA copies/μL. The small intestine had the highest viral copies in the middle stage with 8log10 RNA copies/μL. In addition, the virus could be detected in low quantities in ileums during the late stage while other tissue samples were negative (Fig. 3).

### Histopathology

**Fig. 4:**
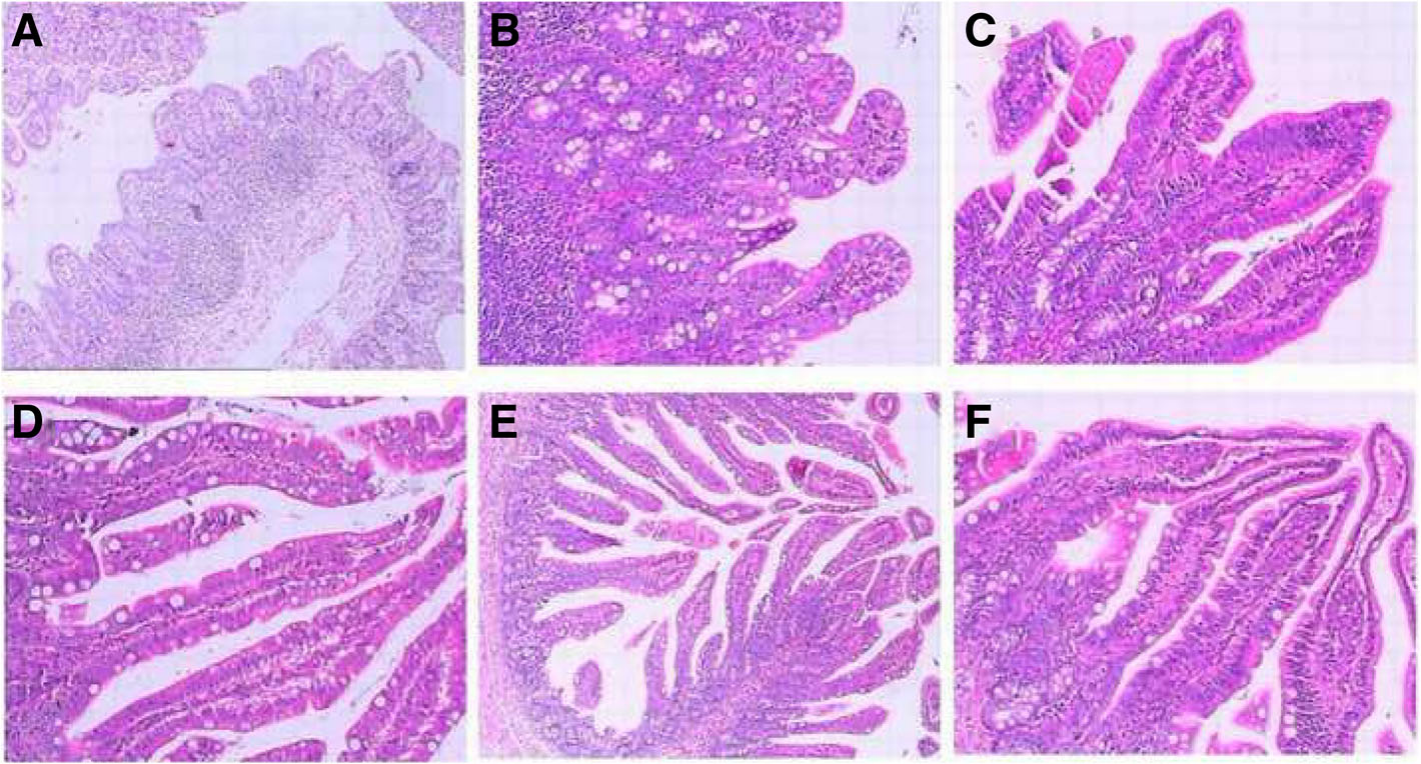
Histologic lesions of ileum caused by PDCoV. (**a**) In the early stage (2 dpi), the ileum slices of the experiment group (× 80); (**b**) In the middle stage(4 dpi), ileum tissues from PDCoV-infected piglets (× 200); (**c**) In the late stage, ileum tissues from PDCoV-infected piglets (× 200); (**d**) In the early stage (2 dpi), ileum tissues from the control group; (**e**) in the middle stage (4 dpi), ileum tissues from the control group (× 80); (**f**) in the late stage, ileum tissues from the control group (× 200)

Histological changes associated with PDCoV infection were observed in duodenum, jejunum, and ileum, but the lesions in the ileum were most obvious. In comparison with small intestine sections of normal nursing piglets, mild to severe villous atrophy was observed in pigs infected with PDCoV at 2 dpi necropsy. There were obvious lesions in the ileum. Histologic lesions included villous blunting, mucosal inflammatory cells increasing, or small intestinal cell shedding and necrosis at 2 dpi (Fig. 4a and d). At 4 dpi, enterocytes often had a vacuolated cytoplasm. Besides, more severe villous atrophy and occasional fusion of villi were observed in the ileums. The lamina propria cells showed symptoms of hyperemia infiltration (Fig. 4b and e). At 11 dpi, the duodenum, jejunum and ileum of the piglets exhibited recovery in different degrees. Microscopic examination of the small intestines of piglets in experimental group showed that the villi was slender, and the small intestine epithelium had the intact nuclei and was closely next to the lamina propria cells. As the same time, macrophages and lymphocytes were well defined and completed (Fig. 4c and f).

### Immunothistochemistry

**Fig. 5:**
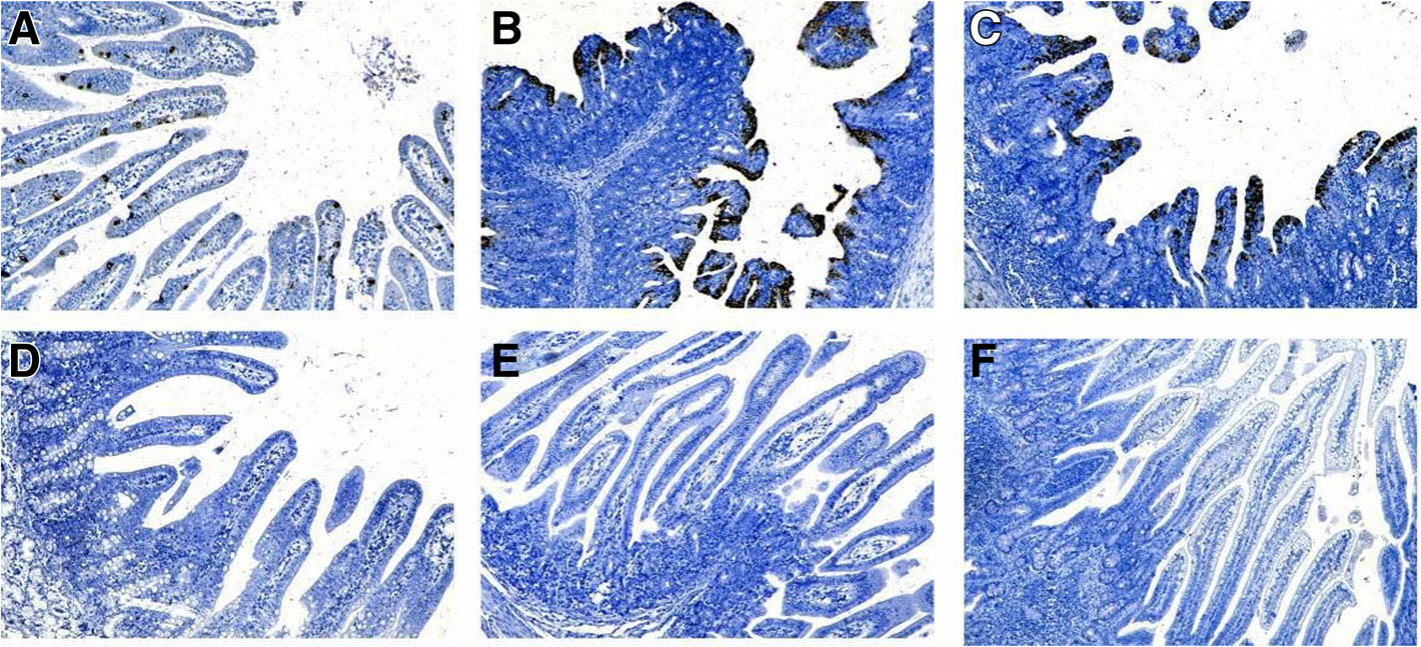
Immunohistochemistry (IHC) analysis of small-intestine sections in the peak of stage. The villous enterocytes of PDCoV-inoculated pigs were stained brown and black by DAB. This phenomenon demonstrated that PDCoV antigen was detected in the villous enterocytes of PDCoV-inoculated pigs at the peak of stage and examples of positive staining are presented in Fig. 5a, b and c. (**a**) the duodenum slides of the experiment group (× 80); (**b**) the jejunum slides of the experiment group (× 80); (**c**) the ileum slides of the experiment group (× 80); (**d**) the duodenum slides of the mock-control group (× 80); (**e**) the jejunum slides of the mock-control group (× 100); (**f**) the ileum slides of the mock-control group (× 140)

As shown in Fig. 5, compared with the mock-control group, the small intestine villous epithelium of the duodenum, jejunum and ileum in the PDCoV-inoculated group, were stained brown and black by DAB in varying degrees during three experimental periods. It demonstrated that PDCoV mainly concentrated at the small intestine villous epithelium which provided a breeding ground for virus’ propagation and replication. In terms of infection, PDCoV mainly aggregated in the duodenum in the early stage, while in the peak of stage and the later period, the virus transferred to ileum. The phenomenon was corresponded with the result of real-time RT-PCR.

### Overview of differentially regulated genes after PDCoV infection

Microarray analysis identified that 20,674 genes were differentially expressed more than three hundred-fold in response to PDCoV infection. During early onset, 1218 genes were up regulated and 1632 genes were down regulated. At the peak of the disease, 698 genes were up regulated and 983 genes were down regulated. During the recovery phase, 174 genes were up regulated and 171 genes were down regulated (Table 2). Overall, these genes were classified into 12, 667 and 7 biological processes in the three infection periods, respectively. The 20 largest biological process groups were involved in lipid metabolism, amino acid metabolism, glucose metabolism, digestion and absorption and related signaling pathways.

**Table 2:** Statistics of differential expression genes between the challenge group and the control group

Peirod	Experimental group	Control group	Up-regulated genes	Down-regulated genes	The total of differential expression genes
Early stage	B1	A1	1218	1632	2850
Middle stage	B2	A2	698	983	1681
Late stage	B3	A3	174	171	345

Horizontal comparisons identified that biological processes enriched in metabolic regulation included single-organism, cellular lipid metabolism, cellular amino acid metabolic processes, organic acid metabolic process and small molecule metabolic processes. However, only 7 Gene Ontology (GO) terms were significantly different between the control and the experimental group in the recovery phase of the infection. The most significant difference was in serine endopeptidase activity.

### Detection of differential expression between the challenge group and the control group

**Fig. 6:**
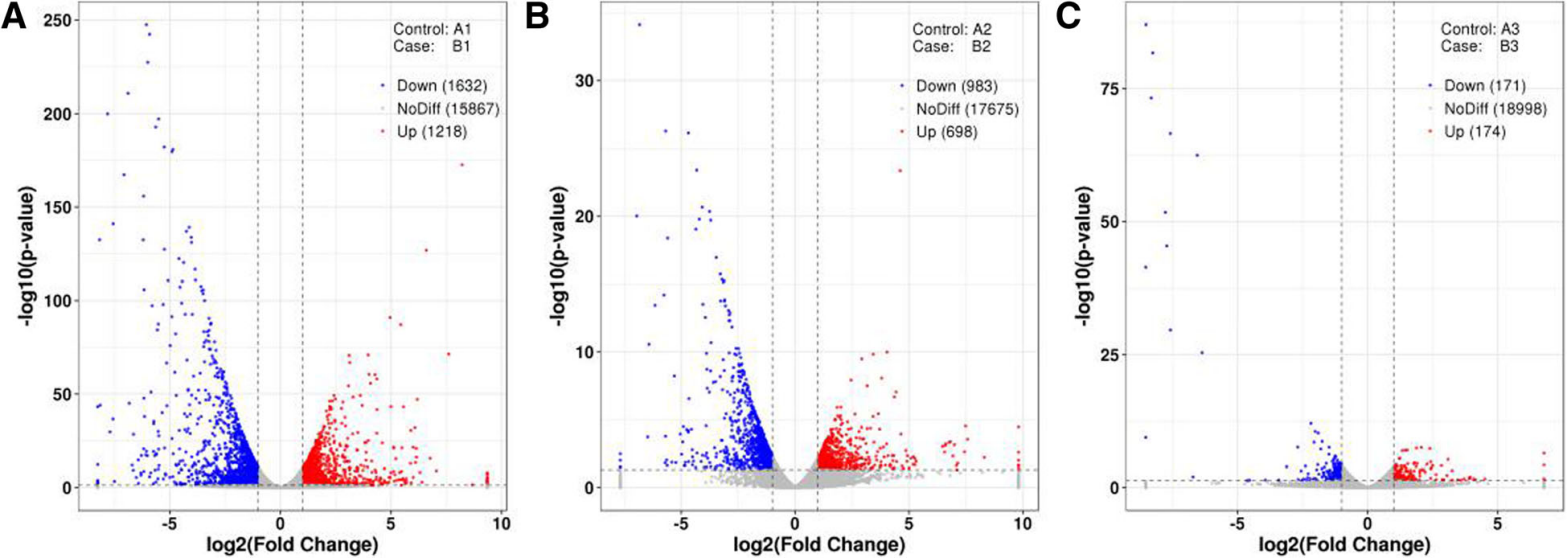
The differently expression genes of volcano picture in three periods. The vertical line is the 2-fold expression difference threshold; the horizontal line is the *P*-value = 0.05 threshold; the red, blue and gray points represent the up-regulated, down-regulated and non-significant differentially expressed genes; **a**, pre-onset; **b**, mid-onset; **c** Recovery period

The threshold for up- and down-regulated genes was a fold change of ≥2.0 and a *P* value ≤0.05. We found that TGM3 was the most highly regulated gene in the early onset, which was changed 654.8-fold. And CYP2B22 was the most down-regulated gene 0.0030-fold in this period. During peak onset, the PI3 gene was significantly up-regulated 895.8-fold and LOC100516628 was down-regulated 0.0033-fold. In the recovery phase, UBTFL1 was up-regulated 22.8-fold while LOC110255257 was down-regulated 0.0027-fold (Fig. 6).

### Verification of microarray data using real-time qRT-PCR

GO analysis (http://www.geneontology.org) and KEGG pathway analysis (http://www.genome.jp/kegg/) were applied to determine the roles of differentially expressed mRNAs. A *p*-value< 0.05 and a false discovery rate of < 0.05 were used as thresholds.

**Fig. 7:**
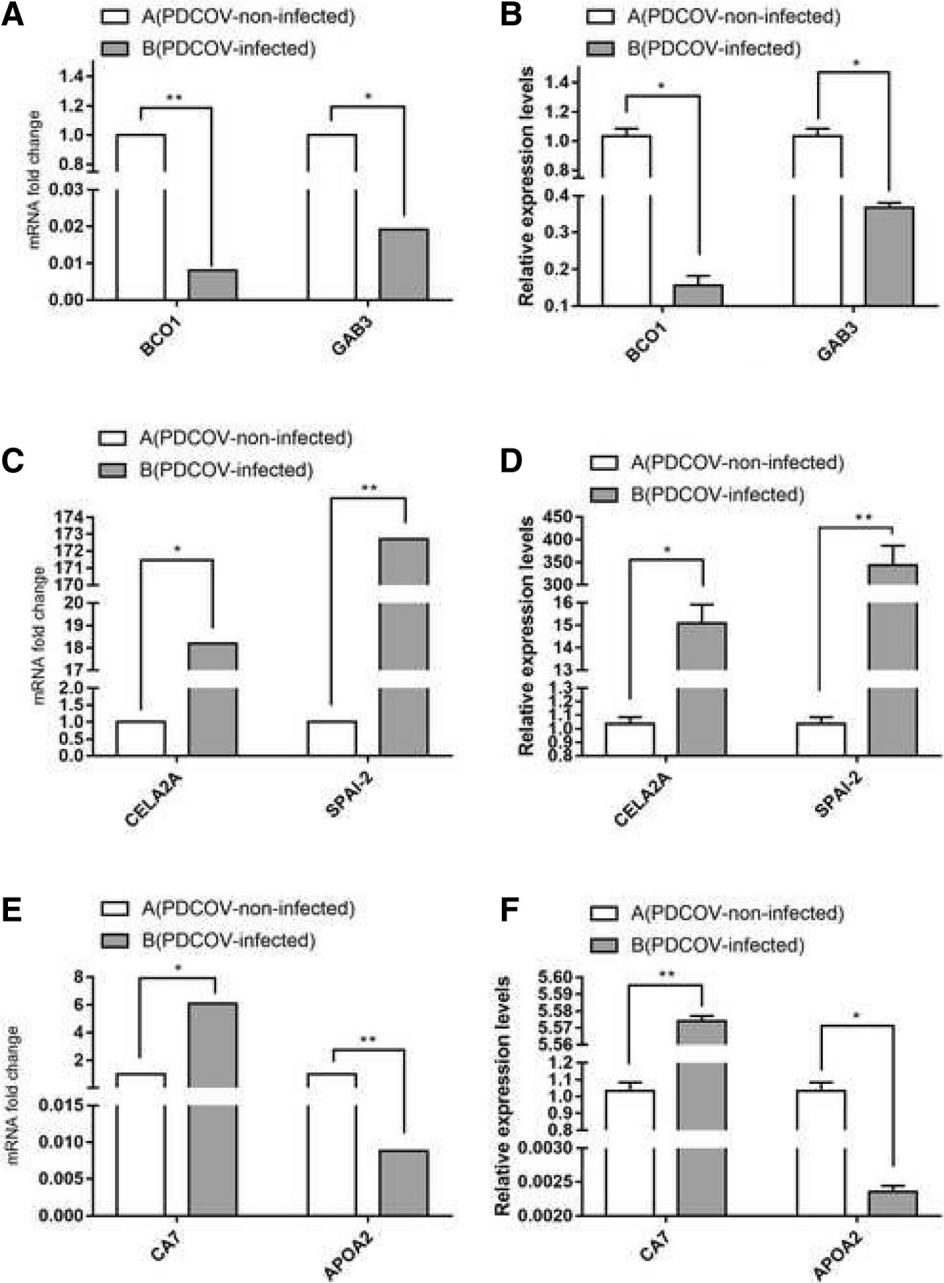
Verification of microarray data. Verification of microarray data using real-time qRT-PCR six genes related to metabolic pathway (BCO1, GAB3, CELA2A, SPAI-2, CAT, APOA2). The data demonstrated that the overall real-time RT-PCR results correlated well with the microarray. **a** analysis of fold change of BCO1 and GAB3 by using microarray; **b** analysis of fold change of BCO1 and GAB3 by using real-time RT-PCR; **c** analysis of fold change of CELA2A and SPAI-2 by using microarray; **d** Analysis of fold change of CELA2A and SPAI-2 by using real-time RT-PCR. **e** analysis of fold change of CA7 and APOA2 by using microarray; **f** Analysis of fold change of CA7 and APOA2 by using real-time RT-PCR

The differentially expressed genes with the highest expression levels in the microarrays were BCO1, GAB3, CELA2A, SPAI-2, CA7 and APOA2. All these genes passed the quality control tests and demonstrated the same relative changes as in the microarray analysis although there were some differences in fold change levels (Fig. 7). These two methods revealed the same relative regulation patterns of mRNA abundance and indicated that many genes were significantly changed in response to PDCoV infection.

### GO term analysis

GO functional enrichment analysis of the differentially expressed genes indicated that 126, 67 and 7 GO terms were significantly enriched in the early, middle and late infection stages, respectively. The top 10 GO terms were enriched in biological processes, cellular components and molecular function. A horizontal comparison of these results indicated that the biological processes were enriched in single-organism catabolic processes, cellular lipid metabolic processes, organic acid metabolic processes and small molecule metabolic processes. The cellular component group were primarily concentrated in the extracellular and plasma membrane regions. The molecular function groups during the early stage of infection were primarily involved in iron and oxidoreductase activity. The middle infection stage revealed transmembrane, cation transmembrane transporter activity and enzyme inhibitor activities. However, the late infection stage showed the least number of differences between experimental and control groups with only 7 GO terms. The serine-type endopeptidase activity was the most significant among the latter.

### KEGG pathway analysis

**Fig. 8:**
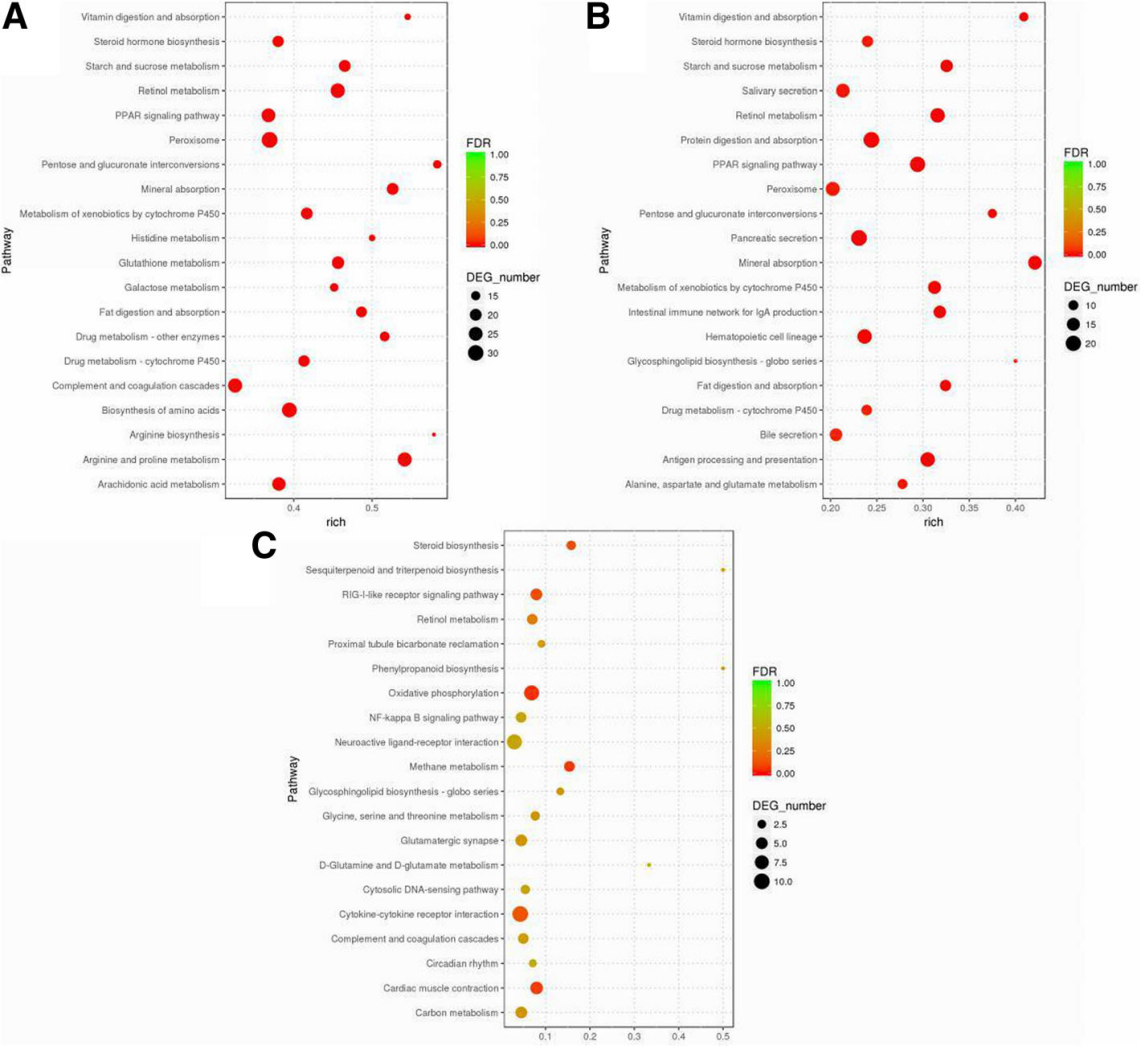
Different KEGG pathway gene enrichment statistics. **a**, pre-infection; **b**, mid-term infection; **c**, late infection; dot diameter indicates the number of differential genes, color depth indicates significance, abscissa indicates enrichment abundance, and ordinate indicates different pathways

KEGG pathway analysis revealed that 43 pathways were significantly altered during PDCoV infection. The predominant pathways involved both catabolic and anabolic metabolism. These involved amino acid metabolism of glutamate, arginine, serine, threonine and aspartic acid as well as starch and sucrose metabolism, pentose conversion and galactose metabolism (Fig. 8). The lipid metabolism pathways involved arachidonic acid, glycerophospholipids and glyceride. Digestion and absorption of minerals, fat, proteins, vitamins and carbohydrates were all significantly changed. Endocrine system alterations included PPAR and adipocytokine signaling as well as immune system changes.

## Discussion

Since PDCoV being identified in 2009 in HongKong, many studies had focused locally on the virus characterization and evolution [1–4]. To fill the gap of interaction between virus and host, particularly the correlation analysis between clinical symptoms and differential expressed host genes, 5-day-old piglets were orally challenged with PDCoV strain, CHN-GD16–05, and used for mRNA expression profile analysis in this study.

Clinical symptoms, diarrhea, vomiting and mental depression, of pigs were observed as early as the third day after the PDCoV challenge. 3/20 pigs died and the others began to recovery after 11 days. The intestine of diarrhea pig after dissection was found to be filled with water and its wall became almost paper-thin. The highest PDCoV load determined by qPCR was located in the ileum, followed by jejunum, duodenum and mesenteric lymph nodes, which differs from PEDV that concentrates in posterior jejunum [16]. But the mechanism of it needs further study. In this study, the differentially expressed FABPs (Fatty acid-binding proteins) genes and diversification of FABPs gene mediated metabolism process were speculated to be responsible for the co-occurrence of the serious destruction of intestinal epithelial and body weight loss in piglets infected with PDCoV. This is somewhat similar to the study of Chen et al. on expression profile analysis of circRNAs in PEDV infected IPEC-J2 cells, a porcine ileum epithelium cell line [17]. The study of Chen et al. demonstrated that PEDV were closely related to the metabolism process of ubiquitin mediated proteolysis [17]. Study of Zhao et al. also indicated that TGEV was responsible for dysfunctional mitochondria in small intestine epithelial cells of piglets via up-regulating ssc-miR-4331 and activating p38 MAPK pathway [18].

FABPs, which are highly expressed in the cytoplasm of various cells in the small intestine, play prominent roles in the PPAR signaling pathway and are related to lipogenesis, lipid metabolism, inflammation, insulin sensitivity as well as cell growth and differentiation [19, 20]. Herein FABPs were found to be regulated by PDCoV. Fatty acid-binding protein 1 (FABP1) and fatty acid-binding protein 3 (FABP3), which not only mediate fatty acid transportation to different cell organelles such as mitochondria, peroxisome, endoplasmic reticulum and nucleus, but also modulate fatty acid metabolism and storage as a signaling molecule outside the cell, attracted our special attention [20].

FABP1 was reported to play a key role in fatty acid storage [21, 22]. Furthermore, fatty acids, particularly arachidonic-acid, can be metabolized into a family of lipid mediators such as eicosanoids, which can function as anti-inflammatory such as cyclopentenone prostaglandins (PGA1, PGA2 and PGJ2) and pro-inflammatory factors [20, 21]. Significant down-expression of FABP1 (fold change =0.083) may result in decreased secretion of anti-inflammatory factors. And increased mucosal inflammatory cells may cause intestinal injury. It is reported that down-regulation of FABP1 expression, significantly associated with poor differentiation and high expression of β-catenin and glutamine synthetase (GS), caused phenotypic changes during tumor progression in almost 10% of hepatocellular carcinoma (HCC) [22–24]. FABP3 was found to participate in mitochondrial energy metabolism by transporting fatty acid during ATP production and promote myocardial cell apoptosis [23–25]. So in this study, sharply up-regulated expression (fold change = 6.8) of FABP3 was speculated to mediate the mitochondrial dysfunction and cell apoptosis of the intestinal epithelial cells, which may be responsible for the emaciation of PDCoV challenged piglets. Therefore, it can be preliminarily concluded that PPAR differential expression caused by PDCoV was mostly associated with weight loss and death from emaciation. The verification of correlation analysis between weight loss and the target genes described above will be on schedule in the near future. Intestinal epithelial cells, which may be responsible for the emaciation of PDCoV challenged piglets. Therefore, it can be preliminarily concluded that PPAR differential expression caused by PDCoV was mostly associated with weight loss and death from emaciation. The verification of correlation analysis between weight loss and the target genes described above will be on schedule in the near future.

## Conclusions

In summary, this is the first report of mRNA expression of 5-day old piglets following PDCoV infection. 20, 675 significant differentially expressed genes, which were associated with infection response, metabolism signaling and organismal systems signaling pathways, were described in this study. Especially, the highly differential expression of FABP genes was speculated to be mostly associated with weight loss and death from emaciation of PDCoV challenged piglets. These results provide new insights into the metabolic mechanisms and pathogenesis of PDCoV infection.

## Abbreviations

ACAA1: Acetyl-CoA acyltransferase 1
ACADL: Acyl-Coenzyme A dehydrogenase, long-chain
ACSL1: Acyl-CoA synthetase long chain family member 1
ACSL3: Acyl-CoA synthetase long chain family member 3
APOA1: Apolipoprotein A1
APOA2: Apolipoprotein A2
APOA2: Apolipoprotein A2LPL
APOC3: Apolipoprotein C3
BCO1: Beta-carotene oxygenase 1
CA7: Carbonic anhydrase 7
CELA2A: Chymotrypsin like elastase family member 2A
CoVs: Coronaviruses
CPE: Cytopathic effects
CPT1C: Carnitine palmitoyltransferase 1C
CSFV: Classical swine fever
CYP2B22: Cytochrome P450 2B22
dpi: days post-infection
EHHADH: Enoyl-Coenzyme A, hydratase/3-hydroxyacyl Coenzyme A dehydrogenase
FABP1: Fatty acid binding protein 1
FABP3: Fatty acid binding protein 3
GAB3: GRB2 associated binding protein 3
GO: Gene ontology
HK: Hong Kong
hpi: hours post-infection
LOC100516628: UDP-glucuronosyltransferase 2B18-like
LOC110255257: oral-facial-digital syndrome 1 protein-like
LPL: Lipoprotein lipase
MMP1: Matrix metallopeptidase 1
MOI: a multiplicity of infection
PBV: Picobirnavirus
PCV3: Porcine circovirus 3
PDCoV: Porcine deltacoronavirus
PEDV: Porcine epidemic diarrhea virus
PEDV: Porcine epidemic diarrhea virus
PI3: Peptidase inhibitor 3
PPAR: Peroxisome proliferator-activated receptor
PRRSV: Porcine reproductive and respiratory syndrome virus
RV: Rotavirus
SCP2: Sterol carrier protein 2
SLC27A4: Solute carrier family 27 member 4
SPAI-2: Sodium/potassium ATPase inhibitor SPAI-2
ST: Swine testicular cells
SVA: Senecavirus A
TGEV: Porcine transmissible gastroenteritis virus
TGEV: Transmissible gastroenteritis virus
TGM3: Transglutaminase 3
UBTFL1: Upstream binding transcription factor, RNA polymerase I-like 1
